# Supplementary material for: All-at-once RNA folding with 3D motif prediction framed by evolutionary information
Source: Nat Methods. 2025 Oct 3;22(10):2094–106. doi: 10.1038/s41592-025-02833-w (PMC12510878; doi:10.1038/s41592-025-02833-w)
Supplement: Supplementary file 3 — Source code to run CaCoFold-R3D. Data and results. [file 41592_2025_2833_MOESM3_ESM.gz › supplemental_material/results/CaCoFold-R3D/CaCoFold-R3D_Rfam/RF02105_DLEU2_1.R2R.sto.pdf]

RF02105_DLEU2_1

5′- Y U A R A C C A A A U Y U A U C U A A A A R U U U A R R R Y A Y U U U U U U Y U C Y Y U U U U R U A U A U U Y A A U G A G A A A Y A A C A C U Y A R A U A A A A C U U U U Y C A U G R R A A G R R A Y R G U A A U A U C C A C
